# From intraosseous meningioma and ossified metaplastic meningioma to osteomeningioma: a novel voxel-based, atlas-normalized MRI framework for the radiological classification of skull-involving meningiomas

**DOI:** 10.1016/j.ynirp.2026.100349

**Published:** 2026-04-25

**Authors:** Benoit Hudelist, Walter Thomas, Angela Elia, Marco Demasi, Alessandro Moiraghi, Petra Bintintan, Elias AL. Helou, Clément Debacker, Kor Gael Toruslu, Gonzague Defrance, Shuroq Taju, Fabrice Chrétien, Catherine Oppenheim, Marc Zanello, Alexandre Roux, Johan Pallud

**Affiliations:** a Department of Neurosurgery, GHU Paris Psychiatrie et Neurosciences, Hôpital Sainte-Anne, Paris, France; b Université Paris Cité, Paris, France; c Inserm, U1266, IMA-Brain, Centre de Psychiatrie et Neurosciences, Paris, France; d Department of Neuroradiology, GHU Paris Psychiatrie et Neurosciences, Hôpital Sainte-Anne, Paris, France; e Department of Neuropathology, GHU Paris Psychiatrie et Neurosciences, Hôpital Sainte-Anne, Paris, France

**Keywords:** En plaque meningioma, Meningioma, Meningioma with osseous metaplasia, Neurosurgery, Osteomeningioma, Voxel-based analysis

## Abstract

**Background and objectives:**

Skull-involving meningiomas remain ill-defined, resulting in heterogeneous classifications and terminology. Existing schemes mainly describe bone-dura relationships and often overlook the relative burden of the intracranial soft-tissue component. This study aimed to develop and apply a deterministic, atlas-normalized, MRI-based radiological framework for the standardized description of imaging-defined osteomeningiomas and to explore associations between compartmental tumor distribution, radiological phenotype, and clinical presentation.

**Methods:**

We retrospectively reviewed adults with skull-involving meningiomas at our tertiary neurosurgical center between 2000 and 2024. Tumors were segmented on contrast-enhanced T1-weighted MRI, normalized to MNI152 space, and classified using a deterministic voxel-based radiological framework across osseous, juxta-osseous/dural, and intradural compartments. Tumors were classified as primary osteomeningioma (POM; isolated osseous compartment involvement) or secondary osteomeningioma (SOM; osseous plus adjacent juxta-osseous/dural compartment involvement), with subtypes SOM-I (no intradural extension), SOM-IIA (all three compartments involved, with an osseous component equal to or greater than the intradural component), and SOM-IIB (all three compartments involved, with an intradural component greater than the osseous component). These imaging-defined categories were intended as radiological descriptors of compartmental tumor distribution rather than distinctions between microscopic osseous invasion, reactive hyperostosis, or osseous metaplasia. All analyses were performed at the tumor level, with a predefined sensitivity analysis restricted to one index tumor per patient. Exploratory multivariable logistic regression models were fitted for brain edema, epileptic seizure, raised intracranial pressure, and exophthalmos.

**Results:**

A total of 168 tumors from 149 patients were analyzed. Distribution was POM in 6 cases (3.6%), SOM-I in 37 cases (22.0%), SOM-IIA in 57 cases (33.9%), and SOM-IIB in 68 cases (40.5%). Convexity predominated in POM but was less common in other subtypes. SOM-IIB had the largest intracranial soft tissue component (29.9 ± 30.2 cm^3^) and the highest rate of brain edema, whereas SOM-IIA had the largest osseous component (24.8 ± 25.9 cm^3^). Epileptic seizures and signs of raised intracranial pressure were most frequent in SOM-IIB, exophthalmos in SOM-I, and subcutaneous mass in POM. In exploratory adjusted analyses, SOM-IIB remained associated with brain edema, epileptic seizure, and raised intracranial pressure, whereas SOM-I remained associated with exophthalmos.

**Conclusions:**

This voxel-based, atlas-normalized MRI framework provides a radiological standardization for the description of skull-involving meningiomas. Rather than establishing histological proof of bone or dural invasion, it standardizes compartmental tumor burden across osseous, juxta-osseous/dural, and intradural spaces. In exploratory analyses, the proposed imaging-defined subtypes were associated with distinct clinicoradiological presentation patterns, which warrant further pathological, multimodal, and external validation.

## Highlights

• A voxel-based framework objectively classifies osteomeningiomas based on MRI.
• Classification aligns with location and soft-tissue versus osseous burden.
• Secondary osteomeningioma IIB aligns with epileptic seizures and raised ICP.
• Secondary osteomeningioma I aligns with exophthalmos.
• Primary osteomeningioma aligns with subcutaneous masses.

## Graphical abstract

**Figure:**
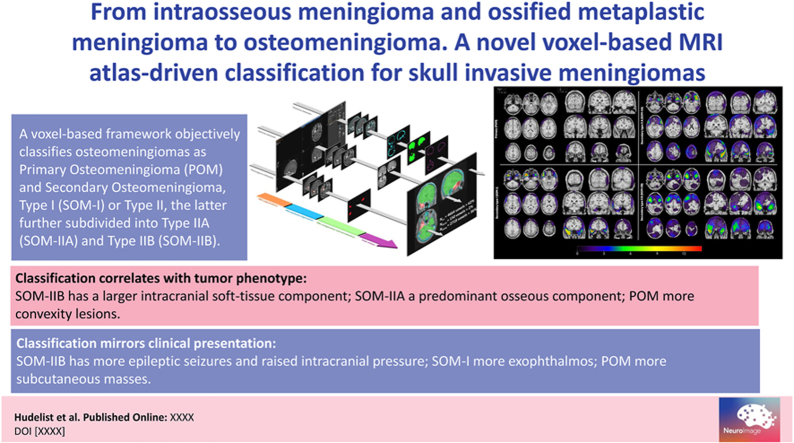

## Abbreviations

5-mFI: 5-item Modified Frailty Index
BIDS: Brain Imaging Data Structure
MNI: Montreal Neurological Institute
NIFTI: Neuroimaging Informatics Technology Initiative
ROI: Region of Interest
POM: Primary Osteomeningioma
SOM: Secondary Osteomeningioma
SOM-I: Secondary Osteomeningioma, Type I
SOM-IIA: Secondary Osteomeningioma, Type IIA
SOM-IIB: Secondary Osteomeningioma, Type IIB

## 1 Introduction

Meningioma is the most frequent intracranial tumor, accounting for approximately 40% of intracranial neoplasms in adults (Ostrom et al., 2021). While most meningiomas are dural-based extra-axial tumors, a subset shows visible osseous involvement on imaging and has been described under heterogeneous labels including en plaque meningiomas, meningiomas with osseous metaplasia, metaplastic meningiomas, or osseous meningiomas (the so-called osteomeningiomas). Due to inconsistencies in terminology across studies, the reported prevalence of osteomeningiomas ranged 4-17% of all intracranial meningiomas (Butscheidt et al., 2019; Li et al., 2024). Their imaging characteristics, classification and even pathogenesis remain matters of debate, with relevant clinical implications (Boetto et al., 2021; Butscheidt et al., 2019; Como et al., 2023; Fountain et al., 2021; McDonald et al., 2025; Pieper et al., 1999). Definitions of osteomeningiomas vary: some authors describe them as purely intraosseous tumors without dural involvement (Butscheidt et al., 2019; Elder et al., 2007; Kwon et al., 2019; Rosahl et al., 2004), whereas others consider them as primarily dural-based meningiomas with secondary invasion of the bone (McDonald et al., 2025; Takase and Yamamoto, 2022).

In the present study, “osteomeningioma” refers to an imaging-defined skull-involving meningioma with a visible enhancing osseous component on contrast-enhanced MRI, regardless of whether the underlying mechanism is true bone invasion, reactive hyperostotic, or osseous metaplasia. Accordingly, our framework is intended as a radiological standardization tool rather than a histopathological distinction between these entities.

Surgical management typically involves the resection of the bone segment infiltrated by tumor, followed by dural resection, and appropriate dural and cranial reconstruction. The objective is to achieve complete removal according to Simpson grade principles (Balasubramanian et al., 2024; Lemée et al., 2019; Nanda et al., 2016; Zuluaga-Garcia et al., 2025). However, recent reports suggest that tumor-associated hyperostotic does not necessarily reflect aggressive invasion, particularly in World Health Organization (WHO) grade I meningiomas, and recurrence rates may not differ significantly between tumors with or without superficial bone involvement (Li et al., 2024). A clearer understanding of the biological behavior and growth pattern of osteomeningiomas is essential to refine surgical strategies, improve patient outcomes, and standardize terminology. Recent advances in neuroimaging and computational methods now enable accurate mapping of tumor distribution using voxel-based methods. Probabilistic, MRI-based brain atlases have already been developed for gliomas (Roux et al., 2019; De Witt Hamer et al., 2013) and meningiomas (Hirayama et al., 2018; Patel et al., 2024; Sun et al., 2020), allowing detailed spatial and statistical analyses that transcend conventional categorical classifications.

The present study aimed to develop a novel voxel-based osteomeningioma classification using a single-center patient cohort, and to evaluate its associations with anatomical phenotype and clinical presentation.

## 2 Materials and methods

### 2.1 Study design and setting

This single-center, retrospective, observational cohort study was conducted at a tertiary referral neurosurgical oncology center between January 2000 and December 2024. Ethical approval was obtained from the institutional review board for human research (IRB00011687; IRB#1: 2024/53). In accordance with French legislation, the requirement to obtain informed consent was waived.

The manuscript was prepared in accordance with the STROBE (Strengthening the Reporting of Observational Studies in Epidemiology) guidelines (von Elm et al., 2007).

### 2.2 Data source

Inclusion criteria were as follows: 1) adult patients (≥18 years); 2) newly diagnosed osteomeningioma suspected on imaging (as defined below) and confirmed by neuropathologic assessment according to the current WHO classification (Louis et al., 2021) and the cIMPACT-NOW update (Sahm et al., 2024) in operated cases; 3) availability of preoperative MRI (minimal slice thickness ≤1.5 mm, isotropic, 3D contrast-enhanced T1-weighted sequence).

### 2.3 Osteomeningioma diagnosis on imaging

All cases were initially diagnosed by a senior neuroradiologist and independently reviewed by two neurosurgeons (B.H. and A.R.). Osteomeningioma was defined radiologically as a skull-involving meningioma with an enhancing osseous component on MRI, with or without adjacent juxta-osseous/dural or intradural extension, characterized on MRI by T1 hypointensity, T2 hyperintensity, and contrast enhancement. This operational imaging definition was designed to capture lesions with visible osseous tumor burden and was not intended to distinguish histologically proven bone invasion from reactive hyperostotic or osseous metaplasia. Isolated hyperostotic without abnormal contrast enhancement or dural thickening was not classified as osteomeningioma. Cases with a suspected alternative diagnosis (including osteoma, fibrous dysplasia, chondrosarcoma, or osteosarcoma) were excluded or designated as indeterminate pending further confirmation.

### 2.4 Cohort ascertainment and diagnostic confirmation

For each tumor, the availability of preoperative CT, surgical treatment, histopathological confirmation, bone specimen availability, and pathological confirmation of osseous invasion were recorded when available. Tumors were classified as imaging-only when neither surgery nor histopathology was available. Non-assessable bone pathology was defined as tumors with available histopathology but without bone specimen available for assessment of osseous invasion.

### 2.5 Data collection

At diagnosis the following variables were recorded: age, sex, modified 5-index frailty index (5-mFI) (Hudelist et al., 2024), clinical presentation (signs of raised intracranial pressure, focal neurological deficit, epileptic seizure, cranial nerve deficit, exophthalmos, subcutaneous mass, or incidental discovery), tumor location (skull base (spheno-orbital, clinoid, orbital roof, sphenoidal jugum sphenoidale (including olfactory groove), tuberculum sellae, cavernous sinus, petrous part of the temporal bone, and clivus) vs. convexity), osteomeningioma classification (reviewed by two neurosurgeons (B.H. and A.R.), with final assignment established by adjudication with a third neurosurgeon (J.P.) when needed), tumor volume (cm^3^, both osseous and intracranial soft tissue components on contrast-enhanced T1-weighted sequence), brain-related edema (hyperintensity on FLAIR sequence), mass effect (defined as compression, displacement, or deformation of lateral ventricles). Imaging acquisition characteristics and quality-control data are summarized in Supplementary data 1.

Missing data are summarized in Supplementary data 2. Complete-case analyses were performed without imputation.

### 2.6 Imaging processing

**Fig. 1:**
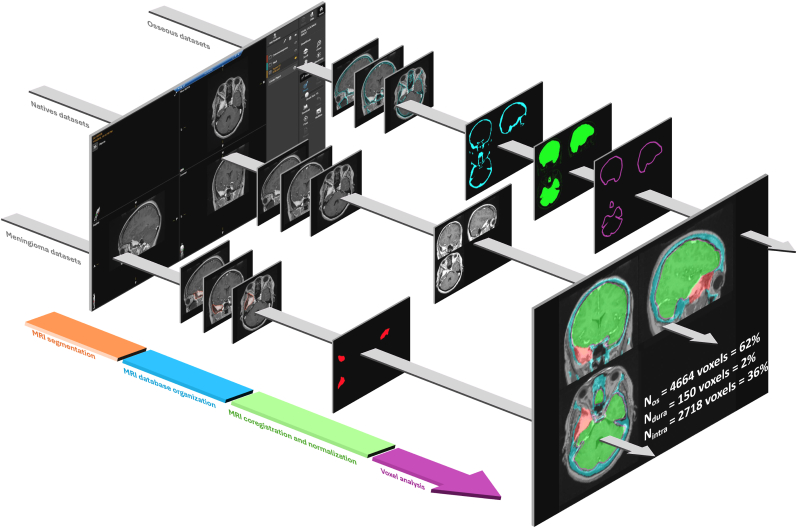
Workflow for tumor-classification image processing. (1) Tumor components on contrast-enhanced T1-weighted sequence were segmented in Brainlab Elements v6.3.0.27 to generate the meningioma mask and the cranial vault (osseous) mask. (2) Native and segmented datasets were exported, converted to NIfTI, and organized according to the BIDS specification. (3) Volumes were coregistered in SPM12 (MATLAB R2021b) and normalized to the MNI152 1-mm template. The skull mask was patient-specific; the dural mask was defined as a 5-mm inward expansion from the internal table. The intradural compartment corresponded to the intracranial space after subtraction of the dural mask. (4) All masks (tumor, skull, dura, and intradural) were resampled into a common space, and each tumor voxel was tested for membership and automatically labeled as osseous, dural, and intradural.

The imaging processing with voxel-based analyses is illustrated in Fig. 1.

*MRI segmentation*. One evaluator (B.H.), blinded to clinical data, management, and follow-up, segmented the meningioma and the cranial vault on preoperative contrast-enhanced T1-weighted sequence using Brainlab Elements 6.3.0.27 (2022 © Brainlab AG, Germany). Two distinct Regions Of Interest (ROI)s were generated: i) the meningioma mask and ii) the osseous mask. To assess the robustness of manual segmentation, a subset of 45 tumors from the same dataset was independently segmented by a second investigator, and inter-rater agreement was evaluated using volumetric correlation and spatial overlap metrics.

*MRI database organization*. Both the segmented and the native sequences were exported separately and converted to Neuroimaging Informatics Technology Initiative (NIFTI) format. The imaging database was structured according to the Brain Imaging Data Structure (BIDS) standard (http://bids. neuroimaging. io).

*MRI coregistration and normalization.* MRI mask was coregistered using open-source software (SPM12, 2014; Functional Imaging Laboratory, Wellcome Trust Center for Neuroimaging, UCL, London) running on MATLAB R2021b (MathWorks, Natick, MA). A deformation field was estimated from the native T1-weighted image to the Clinical Toolbox MNI152 1.0-mm isotropic template (2017, University of South Carolina) using the unified segmentation approach in SPM12. This deformation field was then applied to the segmented ROIs (meningioma and osseous) to resample them into MNI152 space at 1.0-mm isotropic resolution, using nearest-neighbor interpolation without Jacobian modulation. A cost-function masking approach was applied, using the binary tumor mask to improve alignment in lesioned brains (Ripollés et al., 2012).

*Definition of anatomical masks*. Osseous mask was defined as patient-specific skull mask. The so-called dural compartment was defined operationally as a 5-mm-thick juxta-osseous inner-table layer generated by inward expansion from the inner skull surface (Çavdar et al., 2022); it was used as a radiological proximity compartment rather than a true anatomical segmentation of the dura mater. The intradural compartment was defined as the remaining intracranial space after subtraction of this juxta-osseous/dural layer.

*Voxel-based compartment labeling and density*. A mutually exclusive compartment map was created using set operations: osseous = skull; juxta-osseous/dural = 5-mm inner-table layer; intra = intracranial \ juxta-osseous/dural-proximity layer. In case of residual overlaps after resampling, compartment labels were resolved in the order osseous → juxta-osseous/dural-proximity → intradural. For each voxel belonging to the tumor ROI, the voxel inherited the label of the intersected compartment, and voxel-derived metrics were computed per case: absolute counts in each compartment (N_bone_, N_dura_, and N_intra_) and their percentages relative to total tumor voxels. Tumor voxels were thus automatically labeled as osseous, juxta-osseous/dural-proximity, and intradural.

### 2.7 Osteomeningioma classification

**Fig. 2:**
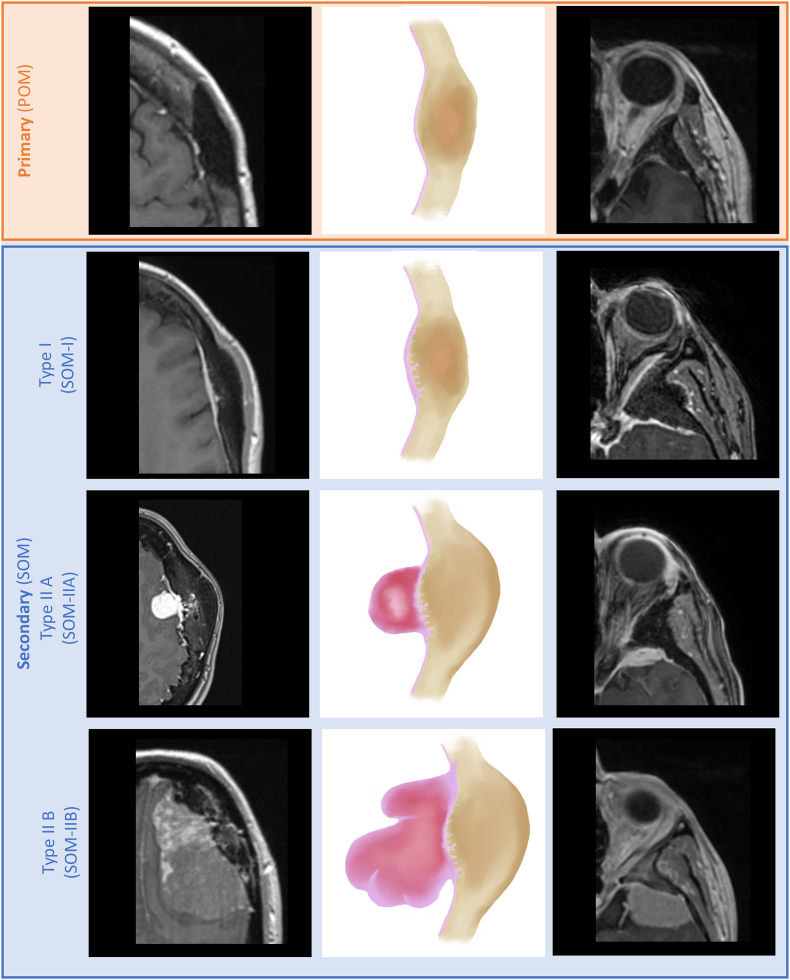
Voxel-based osteomeningioma classification Contrast-enhanced axial MRI sequences (left, cranial vault cases; right, spheno-orbital cases) and corresponding schematic representations (center) illustrate the radiological features of the four subtypes. POM is defined by isolated osseous compartment involvement without juxta-osseous/dural involvement. SOM is defined by osseous plus adjacent juxta-osseous/dural involvement and is subdivided into SOM-I (no intradural extension), SOM-IIA (both osseous and intradural components, with the osseous component equal to or greater than the intradural component), and SOM-IIB (both osseous and intradural components, with the intradural component greater than the osseous component).

Osteomeningiomas were classified radiologically using voxel counts derived from the mutually exclusive osseous, juxta-osseous/dural, and intradural compartments after atlas normalization. For each tumor, labeled voxel counts were computed in each compartment (N_bone_, N_dura_, and N_intra_). The framework was intended to standardize MRI-based compartmental tumor distribution rather than to establish histological proof of invasion. Classification was assigned deterministically as follows: POM, when tumor voxels were confined to the osseous compartment (N_bone_ > 0, N_dura_ = 0, N_intra_ = 0); SOM-I, when osseous and juxta-osseous/dural compartments were involved without intradural extension (N_bone_ > 0, N_dura_ > 0, N_intra_ = 0); SOM-IIA, when all three compartments were involved and the osseous component was equal to or greater than the intradural component (N_bone_ > 0, N_dura_ > 0, N_intra_ > 0, and N_bone_ ≥ N_intra_); and SOM-IIB, when all three compartments were involved and the intradural component exceeded the osseous component (N_bone_ > 0, N_dura_ > 0, N_intra_ > 0, and N_intra_ > N_bone_). In cases of equality between osseous and intradural voxel counts, tumors were classified as SOM-IIA. The classification is illustrated in Fig. 2.

**Fig. 3:**
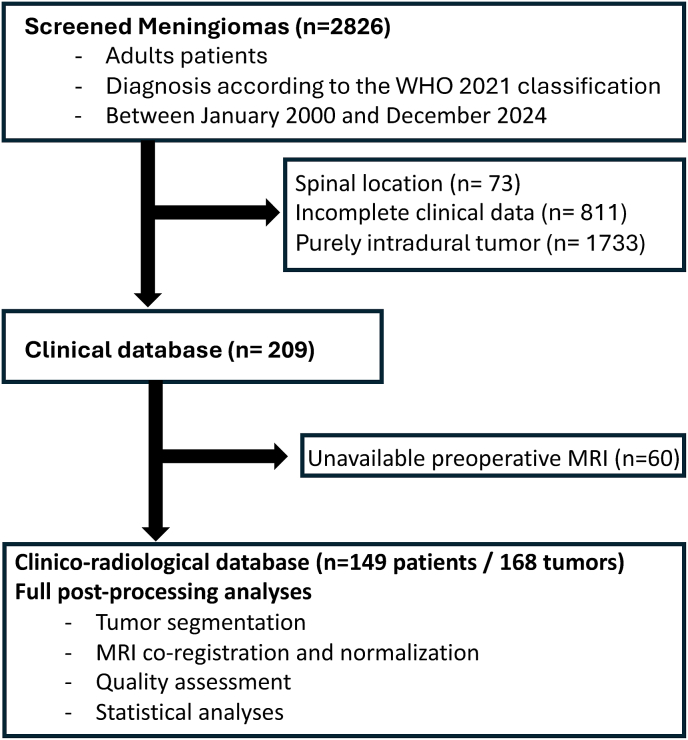
Flowchart shows details of patient selection process.

These categories were defined as imaging-based radiological descriptors of compartmental tumor distribution and were not intended to distinguish microscopic osseous invasion, reactive hyperostosis, or osseous metaplasia.

### 2.8 Statistical analyses

All analyses were performed at the tumor level. Because some patients contributed more than one tumor, we performed a predefined sensitivity analysis restricted to one index tumor per patient, defined as the lesion with the largest total tumor volume. Voxel-derived metrics were computed for each case, including absolute voxel counts in each compartment (N_bone_, N_dura_, and N_intra_) and their percentages relative to total tumor volume. The final osteomeningioma class (POM, SOM-I, SOM-IIA, and SOM-IIB) was treated as a categorical variable. Descriptive statistics were reported as mean ± standard deviation for continuous variables and as frequencies and percentages for categorical variables. Categorical variables were compared using Fisher's exact test, and continuous variables using the Kruskal-Wallis test. These univariable and pairwise subgroup comparisons were considered exploratory and are reported descriptively without formal adjustment for multiple testing. Accordingly, emphasis was placed on effect sizes, confidence intervals, and consistency of direction rather than on nominal p-values alone. To assess whether the main clinicoradiological associations persisted after adjustment, we fitted exploratory multivariable logistic regression models for brain edema, epileptic seizure, signs of raised intracranial pressure, and exophthalmos. Given the limited number of outcome events, models focused on the main subgroup-symptom associations identified in univariable analyses: SOM-IIB versus other classes for brain edema, epileptic seizure, and raised intracranial pressure, and SOM-I versus other classes for exophthalmos. All models were adjusted for age, sex, location (skull base vs convexity), and log-transformed total tumor volume. Results are reported as adjusted odds ratios (aORs) with 95% confidence intervals and should be interpreted as exploratory. Sex-stratified analyses were not performed because the limited number of male patients precluded stable subgroup-specific estimates. To assess potential selection bias, analyzed tumors were compared with non-analyzed cranial osteomeningiomas identified in the institutional database but not retained for voxel-based analysis because of unavailable or non-exploitable preoperative MRI (Supplementary Data 3). Analyses were performed using JMP Pro software (version 18.0.2; SAS Institute Inc., Cary, NC, USA).

## 3 Results

During the study period, 2826 patients underwent management for meningiomas at our institution (see Fig. 3). Of these, 73 were excluded due to spinal location, 1733 due to purely intradural cranial involvement, 811 due to incomplete clinical data, and 60 due to unavailable preoperative MRI. The final cohort comprised 149 patients with 168 osteomeningiomas. Of these, 89 tumors (53.0%) were surgically resected with histopathological confirmation, 50 had preoperative CT available, 26 had bone specimens available, and 79 were classified on imaging only (Supplementary data 4). Bone specimens confirmed osseous invasion in all 26 assessable cases; bone pathology was non-assessable in the remaining 63 resected tumors. These findings provide pathological support for the radiological framework in the subset with available bone specimens, while broader pathological correlation of the osseous component remains to be established.

Inter-rater agreement for the manual segmentation step was evaluated in a subset of 45 tumors independently segmented by two investigators. Volumetric concordance was excellent (Pearson r = 0.987; 95% CI, 0.977-0.993), with high spatial overlap based on Dice and Jaccard indices (Supplementary data 5).

### 3.1 Tumors characteristics

**Fig. 4:**
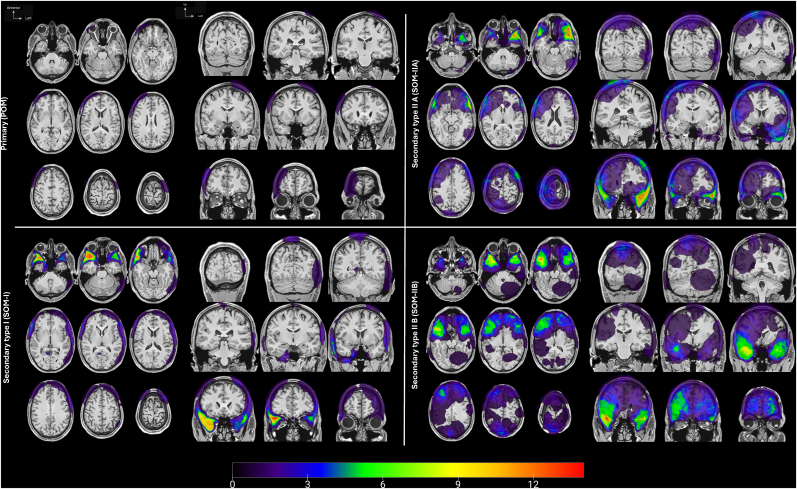
Location and frequency of each voxel-based classification subgroup. Color-coded frequency maps show the spatial distribution and number of cases with meningioma. For each voxel-based classification subgroup, a voxel-based density map is displayed. Images are displayed in radiologic convention.

Patient and osteomeningioma characteristics are summarized in Table 1. The mean age was 56.4 ± 12.5 years, 90% of patients were female. Distribution according to the voxel-based classification was: POM, 6 cases (3.6%); SOM-I, 37 cases (22.0%); SOM-IIA, 57 cases (33.9%); and SOM-IIB, 68 cases (40.5%). Voxel-density maps for each subgroup are shown in Fig. 4. Convexity osteomeningiomas were observed in 85 cases (51%) and were significantly more common in POM (6 cases, 100%) compared with SOM-IIA (30 cases, 53%), SOM-IIB (36 cases, 53%), and SOM-I (13 cases, 36%; p = 0.018). The mean intracranial soft tissue component volume was 16.1 ± 24.0 cm^3^, with the largest volumes observed in SOM-IIB (29.9 ± 30.2 cm^3^) compared with SOM-IIA (10.1 ± 14.1 cm^3^) and SOM-I (2.3 ± 1.9 cm^3^; p < 0.001). The mean osseous component volume was 18.3 ± 20.3 cm^3^, with the largest volumes observed in SOM-IIA (24.8 ± 25.9 cm^3^) compared with POM (22.4 ± 14.0 cm^3^), SOM-I (22.2 ± 19.4 cm^3^), and SOM-IIB (10.4 ± 11.6 cm^3^; p < 0.001). Peritumoral brain edema was present in 65 cases (39%) and occurred more frequently in SOM-IIB (49 cases, 72%) compared with SOM-IIA (14 cases, 26%), or SOM-I (2 cases, 5%; p < 0.001). A mass effect was observed in 44 cases (26%), most often in SOM-IIB (35 cases, 51%) compared with SOM-IIA (8 cases, 14%) and SOM-I (1 cases, 3%; p < 0.001).

**Table 1:** Main characteristics of the study sample (n = 168).

	Whole series (n = 168)	Voxel-based Osteomeningioma Classification				
		POM (n = 6)	SOM-I (n = 37)	SOM-IIA (n = 57)	SOM-IIB (n = 68)	p-value
**Clinical parameters**						
Sex						0.086
Female	151 (90)	5 (83)	36 (97)	53 (93)	57 (84)	
Male	17 (10)	1 (17)	1 (3)	4 (7)	11 (16)	

Age (years), mean (SD)	56.4 (12.5)	52.3 (12.4)	53.8 (9.8)	58.5 (13.9)	56.6 (12.6)	0.341

5mFI ≥2	4 (2)	0 (0)	0 (0)	2 (5)	2 (3)	0.837
**Symptom at diagnosis**						
Incidental	58 (35)	2 (33)	16 (43)	23 (40)	17 (25)	0.220
Neurological focal deficit	32 (19)	0 (0)	9 (24)	10 (18)	13 (19)	0.758
Cranial nerve deficit	21 (13)	0 (0)	8 (22)	9 (16)	4 (6)	0.120
Epileptic seizure	21 (13)	0 (0)	0 (0)	6 (11)	15 (22)	**0.007**
Signs of raised intracranial pressure	27 (16)	1 (17)	1 (3)	4 (7)	21 (31)	**<0.001**
Exophthalmos	30 (18)	0 (0)	14 (38)	11 (19)	5 (7)	**0.001**
Subcutaneous mass	16 (10)	3 (50)	5 (14)	8 (14)	0 (0)	**<0.001**
**MRI characteristics at diagnosis**						
Tumor volume (cm^3^), means (SD)						
Intracranial soft tissue component^a^	16.1 (24.0)	0 (0)	2.3 (1.9)	10.1 (14.1)	29.9 (30.2)	**<0.001**
Osseous component	18.3 (20.3)	22.4 (14.0)	22.2 (19.4)	24.8 (25.9)	10.4 (11.6)	**<0.001**
Tumor volume (cm^3^), median [IQR]						
Intracranial soft tissue component^a^	6.8 [1.9-19.8]	0.0 [0.0-0.0]	1.6 [0.9-3.2]	5.8 [2.2-10.0]	19.6 [10.3-39.3]	**<0.001**
Osseous component	11.9 [5.9-21.9]	23.9 [13.4-26.9]	16.1 [9.4-29.3]	16.5 [8.2-30.2]	7.8 [3.6-14.2]	**<0.001**
Regional location, n (%)						**0.018**
Skull base^b^	83 (49)	0 (0)	24 (64)	27 (47)	32 (47)	
Convexity	85 (51)	6 (100)	13 (36)	30 (53)	36 (53)	
Brain edema						**<0.001**
No	103 (61)	6 (100)	35 (95)	43 (74)	19 (28)	
Yes	65 (39)	0 (0)	2 (5)	14 (26)	49 (72)	
Mass effect^c^						**<0.001**
No	124 (74)	6 (100)	36 (97)	49 (86)	33 (49)	
Yes	44 (26)	0 (0)	1 (3)	8 (14)	35 (51)	

Except where indicated, data are numbers of tumors with percentages in parentheses.

5mFI: Modified 5-Item Frailty Index.

POM: Primary Osteomeningioma.

SOM: Secondary Osteomeningioma.

SOM-I: Secondary Osteomeningioma, Type I.

SOM-IIA: Secondary Osteomeningioma, Type IIA.

SOM-IIB: Secondary Osteomeningioma, Type IIB.

a Including dural enhancement and intradural expansion.

b Spheno-orbital, Clinoid, Orbital roof, Jugum sphenoidale, Tuberculum sellae, Cavernous sinus, Petrous part of the temporal bone, Clivus.

c Defined by compression, displacement, or deformation of the ventricular system.

### 3.2 Presenting symptoms

**Fig. 5:**
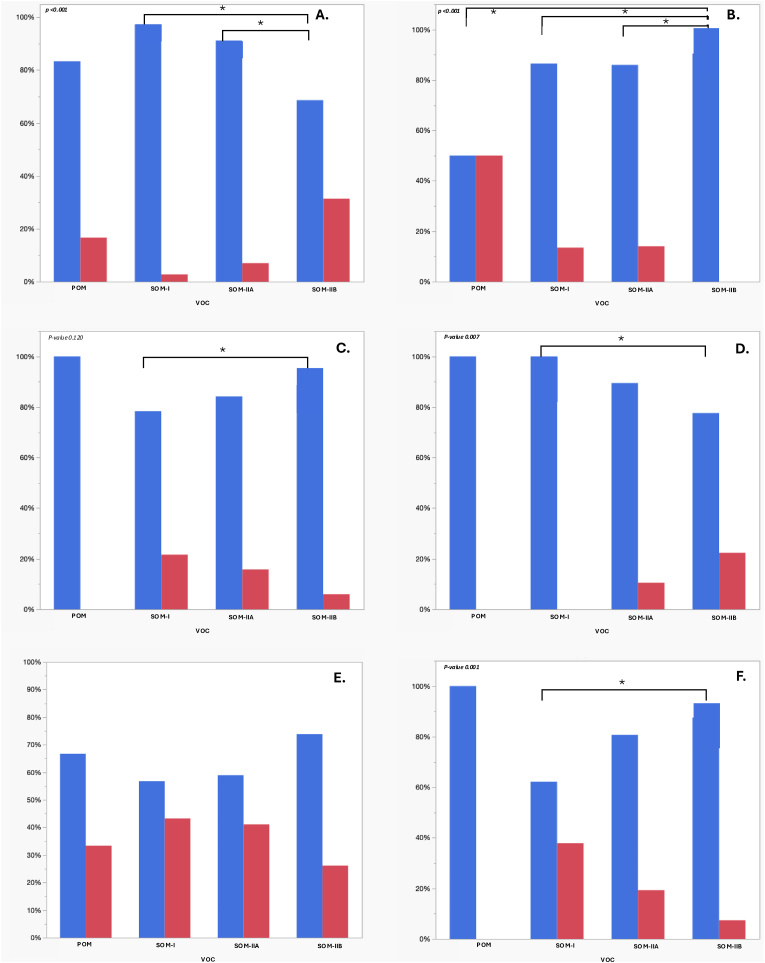
Distribution of key clinical symptoms at diagnosis across the four voxel-based osteomeningioma classification subtypes: POM, SOM-I, SOM-IIA, and SOM-IIB. The six panels represent, respectively: (A) raised intracranial pressure, (B) subcutaneous mass, (C) cranial nerve deficit, (D) epileptic seizures, (E) incidental discovery, and (F) exophthalmos. Each bar plot displays the proportion of cases with or without the symptom in each group. Statistically significant differences between subgroups, based on pairwise Fisher's exact tests, are indicated by asterisks.

Presenting symptoms are detailed in Table 1 and illustrated in Fig. 5. Osteomeningiomas were discovered incidentally in 58 tumors (35%), after presentation with a focal neurological deficit in 32 cases (19%), and following a cranial nerve deficit in 21 cases (13%). No significant differences were observed between classes when comparing global symptomatic versus incidental presentations. Epileptic seizures occurred in 21 cases (13%) and were more frequent in SOM-IIB (15 cases, 22%) compared with POM (0 cases, 0%), SOM-I (0 cases, 0%), and SOM-IIA (6 cases, 11%; p = 0.007). Signs of raised intracranial pressure were present in 27 cases (16%), most often in SOM-IIB (21 cases, 31%) compared with POM (1 case, 17%), SOM-I (1 case, 3%), or SOM-IIA (4 cases, 7%; p < 0.001). Exophthalmos was observed in 30 cases (18%) and more frequent in SOM-I (14 cases, 38%) compared with POM (0 cases, 0%), SOM-IIA (11 cases, 19%), and SOM-IIB (5 cases, 7%; p = 0.001). A subcutaneous mass was present in 16 cases (10%), most common in POM (3 cases, 50%) than in SOM-I (5 cases, 14%), SOM-IIA (8 cases, 14%), and SOM-IIB (0 cases, 0%; p < 0.001).

Pairwise comparisons of clinical presentation by classification subgroup are shown in Fig. 5 and in Supplementary data 6 and should be interpreted as exploratory. Cranial nerve deficits were more frequent in SOM-I than in SOM-IIB (p = 0.024). Epileptic seizures were more frequent in SOM-IIB than in SOM-I (p = 0.001), whereas the comparison between SOM-IIA and SOM-IIB did not reach nominal significance (p = 0.098). Signs of raised intracranial pressure were more frequent in SOM-IIB than in SOM-I (p < 0.001) and in SOM-IIB than in SOM-IIA (p = 0.001). Exophthalmos was more frequent in SOM-I than in SOM-IIB (p < 0.001), whereas the comparison between SOM-I and SOM-IIA did not reach nominal significance (p = 0.058). Subcutaneous mass was more frequent in POM than in SOM-IIB (p < 0.001), in SOM-I than in SOM-IIB (p = 0.005), and in SOM-IIA than in SOM-IIB (p = 0.001).

In exploratory multivariable logistic regression analyses (Table 2), SOM-IIB versus other classes remained independently associated with brain edema (aOR 25.02, 95% CI 9.42-77.19; p < 0.001), epileptic seizure (aOR 4.18, 95% CI 1.54-12.70; p = 0.005), and signs of raised intracranial pressure (aOR 8.83, 95% CI 3.31-27.16; p < 0.001), after adjustment for age, sex, skull base location, and log-transformed total tumor volume. SOM-I versus other classes remained independently associated with exophthalmos (aOR 5.49, 95% CI 1.80-18.76; p = 0.003).

**Table 2:** Exploratory multivariable logistic regression analyses for brain edema, epileptic seizure, raised intracranial pressure, and exophthalmos (n = 168).

Parameter	Adjusted Odds Ratio for Brain edema			Adjusted Odds Ratio for epileptic seizure			Adjusted Odds Ratio for raised intracranial pressure			Adjusted Odds Ratio for exophthalmos^d^		
	aOR^a^	95%CI	p-value	aOR^a^	95%CI	p-value	aOR^a^	95%CI	p-value	aOR^a^	95%CI	p-value
**Sex**												
Female	1 (ref)	-	-	1 (ref)	-	-	1 (ref)	-	-	1 (ref)	-	-
Male	0.59	0.13-2.82	0.500	0.92	0.18-3.55	0.909	NE^c^	unstable	NE^c^	NE^c^	unstable	NE^£^
**Age**	1.00	0.97-1.04	0.872	1.03	0.99-1.07	0.150	1.01	0.97-1.05	0.620	0.96	0.92-1.01	0.120
**Skull base location**												
No	1 (ref)	-	-	1 (ref)	-	-	1 (ref)	-	-	1 (ref)	-	-
Yes	1.41	0.56-3.68	0.466	0.68	0.24-1.83	0.449	0.37	0.13-0.96	0.040	68.32	11.58-1355.44	<0.001
**Log (Total volume + 1)**^b^	6.69	3.58-14.04	**<0.001**	1.48	0.86-2.63	0.170	1.86	1.10-3.33	0.030	2.72	1.38-6.06	0.008
**Voxel-based classification**												
POM vs others	-	-	-	-	-	-	-	-	-	-	-	-
SOM-I vs others	-	-	-	-	-	-	-	-	-	5.49	1.80-18.76	**0.003**
SOM-IIA vs others	-	-	-	-	-	-	-	-	-	-	-	-
SOM-IIB vs others	25.02	9.42-77.19	**<0.001**	4.18	1.54-12.70	**0.005**	8.83	3.31-27.16	**<0.001**	-	-	-

a Variables entered in the multivariable logistic regression models were age, sex, skull base location, log-transformed total tumor volume, and the voxel-based classification term of interest defined a priori for each outcome. SOM-IIB versus other classes was used for brain edema, epileptic seizure, and raised intracranial pressure, whereas SOM-I versus other classes was used for exophthalmos.

b Total tumor volume was entered as log(total volume + 1) because volume distributions were markedly right skewed; adding 1 avoided undefined values for observations with a value of 0. The corresponding odds ratio reflects a 1-unit increase in log-transformed total volume.

c Sex estimates for raised intracranial pressure and exophthalmos were unstable because of sparse data/quasi-separation and should be interpreted with caution.

d Some estimates in the exophthalmos model, particularly sex and skull base location, were unstable because of sparse data/quasi-separation and should be interpreted with caution.

### 3.3 Sensitivity analyses

In predefined sensitivity analyses, the main associations remained directionally consistent when analyses were restricted to one index tumor per patient and to histopathologically confirmed tumors (Supplementary Data 7 and 8). Analyzed and non-analyzed cranial osteomeningiomas were comparable across available baseline characteristics, except for an earlier diagnosis period among non-analyzed tumors (Supplementary Data 3). No significant differences in subgroup distribution or major clinico-radiological variables were observed according to MRI field strength (Supplementary Data 9).

In univariable analyses, SOM-IIB versus all other classes combined was associated with brain edema (OR 13.54, 95% CI 6.38-28.74), epileptic seizure (OR 4.43, 95% CI 1.62-12.11), and signs of raised intracranial pressure (OR 7.00, 95% CI 2.65-18.51). SOM-I versus all other classes combined was associated with exophthalmos (OR 4.38, 95% CI 1.88-10.19) (Supplementary Data 10).

Sensitivity analysis across 2-mm, 3-mm, and 5-mm juxta-osseous/dural-proximity layers showed broadly stable subtype distribution, with 146/168 tumors (86.9%) retaining the same subtype across all three thresholds (Supplementary Data 11). Reclassifications were limited to adjacent categories, mainly from SOM-I to SOM-IIA and, less frequently, from SOM-IIA to SOM-IIB as the layer became thinner; no POM or SOM-IIB tumors changed classification.

## 4 Discussion

### 4.1 Key results

In this single-center, retrospective, observational cohort study of 168 skull osteomeningiomas, we propose a voxel-based MRI classification that quantifies radiological compartmental tumor burden across osseous, juxta-osseous/dural, and intradural spaces. These categories should be interpreted as imaging-defined radiological descriptors rather than as distinct biological entities or surrogates of microscopic osseous invasion. Rather than attempting histological proof of bone or dural invasion, this framework provides a deterministic imaging-based description of lesion distribution. This framework: 1) provides a deterministic voxel-based radiological standardization for osteomeningioma characterization; 2) was associated, in exploratory analyses, with distinct anatomical patterns, including a larger intracranial soft-tissue component in SOM-IIB, a predominant osseous component in SOM-IIA, and a higher frequency of convexity location in POM; and 3) was associated, in exploratory analyses, with distinct clinical presentation patterns, with epileptic seizures and signs of raised intracranial pressure predominating in SOM-IIB, exophthalmos in SOM-I, and subcutaneous mass in POM.

### 4.2 Interpretation

Although osteomeningiomas have long been recognized (HUGHES, 1948; Preedman and Forster, 1948), their origins, management, and biological behavior remain uncertain, which may account for their higher five-year recurrence rate (about 20%) compared with intradural meningiomas (6-9%) (Apra et al., 2020; Li et al., 2024; Liu et al., 2015; Maiuri et al., 2020; Nguyen et al., 2022; Pessina et al., 2019). Bone invasion appears radiologically as tumor-associated hyperostotic and histopathologically as meningioma cell infiltration within trabeculae, often with osteoblastic or osteoclastic reactions (Butscheidt et al., 2019; Pieper et al., 1999). In addition, secreted factors may stimulate osteogenesis without direct invasion, producing bone formation and sclerosis (Di Cristofori et al., 2018; Johnson et al., 2009; McDonald et al., 2025). Thus, hyperostotic likely reflects both direct infiltration and paracrine remodeling (McDonald et al., 2025), explaining the heterogeneous radiological patterns observed (Butscheidt et al., 2019; Pieper et al., 1999). Accordingly, although these mechanisms provide important biological context, the present framework was not designed to resolve them and should not be interpreted as distinguishing true osseous invasion from reactive hyperostosis, osseous metaplasia, or other osteodural remodeling processes on conventional MRI alone.

Terminology remains inconsistent, limiting comparisons across studies. Some authors defined osteomeningiomas as purely intraosseous tumors (primary extradural meningiomas), without dural involvement in 10-30% of cases (Bassiouni et al., 2006; Wang et al., 2024). In voxel-based terms, these correspond to POM or SOM-I. Lang et al. (2000) proposed a CT-based classification by cranial position, later modified by Bassiouni et al. (2006), but neither system accounted for intracranial extension. Liu et al. (2015) analyzed 231 cases (19 local, 212 previously published) and found most were convexity lesions presenting as subcutaneous masses, with 90% benign and a 22.4% recurrence rate. Our voxel-based framework refines this by integrating dural and intradural compartments. SOM-IIB, characterized by substantial intradural expansion, was associated with a greater neurological burden, including more seizures, raised intracranial pressure, and brain edema. In contrast, SOM-IIA, SOM-I, and POM were more often dominated by osseous involvement and frequently presented with cosmetic or orbitofacial manifestations. These findings support the clinical relevance of quantifying compartmental tumor burden, but their implications for treatment timing and surveillance strategies should be evaluated in dedicated prospective studies. Studies focusing on osteomeningiomas with intracranial extension provide additional insights. McDonald et al. (2025) compared 66 hyperostotic meningiomas with 115 non-hyperostotic cases and found that hyperostotic tumors are region-specific, associated with higher intraoperative complication rates, and display distinct radiographic and transcriptional profiles with upregulation of osteogenic factors. However, these results may not apply uniformly across all osteomeningioma subtypes.

Previous classifications of meningiomas with osseous involvement remain heterogeneous and non-standardized. Our atlas-normalized voxel-based framework provides a deterministic description of compartmental tumor distribution, including the intracranial soft-tissue component, which is often clinically relevant. Its main contribution is therefore methodological: to provide an atlas-normalized, voxel-based radiological standardization framework for quantifying relative osseous, juxta-osseous/dural, and intradural tumor burden. It should not be interpreted as a validated biological or histopathological subclassification.

The marked female predominance of the cohort limits generalizability to male patients; although sex was included as an adjustment variable in all multivariable models, the small number of male cases precluded meaningful sex-stratified analyses.

### 4.3 Generalizability

This study provides a standardized image-based definition of osteomeningiomas that may help harmonize terminology across future radiological and clinical studies. Its main strengths are the use of a deterministic atlas-normalized voxel-based classification in a relatively large cohort, the identification of distinct radiological distribution patterns, and the observation that these subtypes were associated with different clinical presentations in exploratory analyses, particularly neurological symptoms. However, this framework should be considered a radiological standardization tool rather than histological proof of bone or dural invasion.

The main associations were preserved in a predefined index-tumor sensitivity analysis, suggesting that within-patient clustering had limited influence on the overall clinicoradiological patterns observed here (Supplementary Data 7). Similar trends were also observed in the histopathologically confirmed subgroup (Supplementary Data 8), which supports the internal consistency of the radiological framework. However, this subset should not be interpreted as providing comprehensive pathological validation of the osseous component, because bone sampling was available in only a minority of resected tumors.

### 4.4 Limitations

This study has several limitations, including its retrospective single-center design, the exploratory nature of the inferential analyses, the absence of formal multiplicity adjustment for secondary subgroup comparisons, and the lack of an external validation cohort. Accordingly, subgroup and pairwise comparisons should be interpreted as hypothesis-generating, with greater emphasis placed on the magnitude and consistency of associations than on isolated nominal p-values. Although imaging acquisition characteristics were documented and no case was excluded because of failed segmentation or spatial normalization, MR acquisition remained heterogeneous over the long study period. The manual segmentation step showed excellent agreement in a 45-tumor inter-rater subset; however, residual scanner- and era-related variability cannot be fully excluded. Reassuringly, the distribution of classification subgroups and the main clinico-radiological variables did not differ significantly according to MRI field strength (1.5T vs 3T) (Supplementary Data 9). Another limitation is that the classification relies on standard contrast-enhanced MRI and therefore cannot reliably distinguish histologically proven osseous invasion from reactive hyperostotic, osseous metaplasia, or other remodeling phenomena at the osteodural interface. In addition, some cases were included on imaging grounds alone, and pathological assessment of bone involvement was not uniformly available across this long retrospective period. Imaging-pathology concordance for osseous involvement could therefore only be assessed in a subset of tumors. Selection bias cannot be fully excluded. However, analyzed and non-analyzed cranial osteomeningiomas were comparable across available baseline variables, with the main difference being an earlier diagnosis period among non-analyzed cases, likely reflecting lower availability of exploitable preoperative MRI in older cases. Finally, formal inter-rater reproducibility of the final POM/SOM subtype assignment was not assessed. Although the manual segmentation step showed excellent agreement in a subset of 45 tumors, independent pre-adjudication subtype assignments were not prospectively archived, precluding retrospective calculation of subtype-level agreement statistics. Formal subtype-level reproducibility therefore remains to be established in future validation studies. The marked female predominance of the cohort also limits generalizability to male patients; although sex was included as an adjustment variable in all multivariable models, the small number of male cases precluded meaningful sex-stratified analyses.

## 5 Conclusion

The proposed voxel-based, atlas-normalized MRI framework provides a deterministic radiological standardization of skull-involving meningiomas based on compartmental tumor distribution. The resulting POM/SOM subtypes should be understood as imaging-defined descriptors rather than as biological or pathological entities. In this retrospective single-center cohort, these radiological subtypes were associated with distinct clinic-radiological patterns in exploratory analyses, but formal subtype-level reproducibility, broader pathological validation, and external confirmation remain necessary before wider adoption.

## CRediT authorship contribution statement

**Benoit Hudelist:** Writing – review & editing, Writing – original draft, Methodology, Investigation, Formal analysis, Data curation. **Walter Thomas:** Writing – review & editing, Writing – original draft. **Angela Elia:** Writing – review & editing, Writing – original draft. **Marco Demasi:** Writing – review & editing, Writing – original draft. **Alessandro Moiraghi:** Writing – review & editing, Writing – original draft. **Petra Bintintan:** Writing – review & editing, Writing – original draft. **Elias AL. Helou:** Writing – review & editing, Writing – original draft. **Clément Debacker:** Writing – review & editing, Writing – original draft. **Kor Gael Toruslu:** Writing – review & editing, Writing – original draft. **Gonzague Defrance:** Writing – review & editing, Writing – original draft. **Shuroq Taju:** Writing – review & editing, Writing – original draft. **Fabrice Chrétien:** Writing – review & editing, Writing – original draft. **Catherine Oppenheim:** Writing – review & editing, Writing – original draft. **Marc Zanello:** Writing – review & editing, Writing – original draft. **Alexandre Roux:** Writing – review & editing, Writing – original draft. **Johan Pallud:** Writing – review & editing, Writing – original draft, Visualization, Validation, Methodology, Formal analysis, Conceptualization.

## Consent to participate

In accordance with French legislation and institutional review board approval, the requirement for informed consent was waived for this retrospective study.

## Ethical approval

Ethical approval was obtained from the institutional review board for human research (IRB00011687; IRB#1: 2024/53). In accordance with French legislation, the requirement to obtain informed consent was waived.

## Funding and disclosures

This research did not receive any specific grant from any funding agency in the public, commercial or not-for-profit sector.

The authors have nothing to disclose.

## Declaration of competing interest

The authors declare that they have no known competing financial interests or personal relationships that could have appeared to influence the work reported in this paper.

## Data Availability

Data will be made available on request.
