# Supplementary material for: From intraosseous meningioma and ossified metaplastic meningioma to osteomeningioma: a novel voxel-based, atlas-normalized MRI framework for the radiological classification of skull-involving meningiomas
Source: Neuroimage Rep. 2026 Apr 25;6(2):100349. doi: 10.1016/j.ynirp.2026.100349 (PMC13134027; doi:10.1016/j.ynirp.2026.100349)
Supplement: Multimedia component 1 [file mmc1.docx]

**Supplementary data 1.** Imaging acquisition characteristics, sequence availability, and quality-control summary.

|  | **All tumors (n=168)** | **POM (n=6)** | **SOM-I (n=37)** | **SOM-IIA (n=57)** | **SOM-IIB (n=68)** |
| --- | --- | --- | --- | --- | --- |
| **Imaging available** |  |  |  |  |  |
| Contrast-enhanced 3D T1 | 168 (100) | 6 (100) | 37 (100) | 57 (100) | 68 (100) |
| FLAIR | 168 (100) | 6 (100) | 37 (100) | 57 (100) | 68 (100) |
| T2 | 168 (100) | 6 (100) | 37 (100) | 57 (100) | 68 (100) |
| CT | 50 (30) | 2 (33) | 11 (30) | 19 (33) | 18 (26) |
|  |  |  |  |  |  |
| **Field strength** |  |  |  |  |  |
| 1.5T | 114 (68) | 5 (83) | 28 (76) | 40 (70) | 41 (60) |
| 3T | 46 (27) | 1 (17) | 9 (24) | 14 (25) | 22 (32) |
| Unknown | 8 (5) | 0 (0) | 0 (0) | 3 (5) | 5 (7) |
|  |  |  |  |  |  |
| **MRI acquisition period** |  |  |  |  |  |
| 2000-2009 | 9 (5) | 0 (0) | 2 (6) | 3 (6) | 5 (8) |
| 2010-2016 | 57 (34) | 2 (33) | 7 (19) | 24 (42) | 24 (35) |
| 2017-2024 | 102 (61) | 4 (67) | 29 (78) | 30 (53) | 39 (57) |
|  |  |  |  |  |  |
| **In-plane voxel size (mm), median [IQR]** | 0.49 [0.47-0.49] | 0.49 [0.49-0.49] | 0.49 [0.47-0.49] | 0.49 [0.47-0.49] | 0.49 [0.47-0.49] |
|  |  |  |  |  |  |
| **Slice thickness (mm), median [IQR]** | 1.35 [1.08-1.40] | 1.40 [1.40-1.40] | 1.25 [1.11-1.40] | 1.20 [1.15-1.40] | 1.40 [1.05-1.40] |
|  |  |  |  |  |  |
| **Excluded for failed segmentation or normalization** | 0 (0) | 0 (0) | 0 (0) | 0 (0) | 0 (0) |

**Supplementary data 2.** Completeness of clinical, radiological, and pathological variables and handling of missing data.

| **Variable** | **Available n/N (%)** | **Missing n/N (%)** | **Handling** |
| --- | --- | --- | --- |
| **Demographic and baseline clinical variables** |  |  |  |
| Age at diagnosis | 168/168 (100.0%) | 0/168 (0.0%) | Complete-case |
| Sex | 168/168 (100.0%) | 0/168 (0.0%) | Complete-case |
| Modified 5-item Frailty Index (5mFI) | 130/168 (77.4%) | 38/168 (22.6%) | Descriptive only / complete-case when used |
| Mode of discovery | 164/168 (97.6%) | 4/168 (2.4%) | Complete-case |
| Signs of raised intracranial pressure | 167/168 (99.4%) | 1/168 (0.6%) | Complete-case |
| Focal neurological deficit | 167/168 (99.4%) | 1/168 (0.6%) | Complete-case |
| Cranial nerve deficit | 167/168 (99.4%) | 1/168 (0.6%) | Complete-case |
| Epileptic seizure | 167/168 (99.4%) | 1/168 (0.6%) | Complete-case |
| Regional location | 168/168 (100.0%) | 0/168 (0.0%) | Complete-case |
|  |  |  |  |
| **Radiological variables** |  |  |  |
| Intracranial soft-tissue component volume | 168/168 (100.0%) | 0/168 (0.0%) | Complete-case |
| Osseous component volume | 168/168 (100.0%) | 0/168 (0.0%) | Complete-case |
| Peritumoral oedema on FLAIR | 168/168 (100.0%) | 0/168 (0.0%) | Complete-case |
| Mass effect | 168/168 (100.0%) | 0/168 (0.0%) | Complete-case |
|  |  |  |  |
| **Imaging acquisition variables** |  |  |  |
| Contrast-enhanced 3D T1-weighted MRI | 168/168 (100.0%) | 0/168 (0.0%) | By inclusion criterion |
| FLAIR MRI | 168/168 (100.0%) | 0/168 (0.0%) | By inclusion criterion / complete-case |
| Preoperative CT status (yes/no) | 168/168 (100.0%) | 0/168 (0.0%) | Descriptive only |
| Field strength | 160/168 (95.2%) | 8/168 (4.8%) | Descriptive only / sensitivity analyses |
| MRI acquisition period | 165/168 (98.2%) | 3/168 (1.8%) | Descriptive only / sensitivity analyses |
|  |  |  |  |
| **Pathology-related variables** |  |  |  |
| Histopathological subtype | 89/168 (53.0%) | 79/168 (47.0%) | Descriptive only; interpretable in operated subset |
| WHO grade | 78/168 (46.4%) | 90/168 (53.6%) | Descriptive only; interpretable in operated subset |
| Bone specimen availability | 26/168 (15.5%) | 143/168 (84.5%) | Descriptive only; interpreted cautiously |
| Bone invasion on pathology | 26/168 (15.5%) | 143/168 (84.5%) | Descriptive only; limited to sampled bone specimens |
| *Completeness was assessed in the final analytic cohort of 168 tumors from 149 patients, and not in the initial screening cohort. Pre-inclusion exclusions due to incomplete clinical data (n=811) or unavailable preoperative MRI (n=60) are reported separately in the cohort selection process and should not be interpreted as within-cohort missingness. Analyses were performed using a complete-case approach on a per-analysis basis, with no formal imputation. Apparent missingness for pathology-related variables partly reflects the fact that only a subset of tumors was surgically treated and that bone sampling was available in only a minority of cases.* | | | |

**Supplementary data 3.** Comparison of analyzed versus non-analyzed cranial osteomeningiomas.

| **Variable** | **Analyzed tumors**  **(n=168)** | **Non-analyzed tumors (n=77)** | **p-value** |
| --- | --- | --- | --- |
| Female sex | 151 (89.9%) | 65 (84.4%) | 0.286 |
| Age at diagnosis, median [IQR] | 54 [49-65] | 53 [48-67] | 0.880 |
| Year of diagnosis, median [IQR] | 2019 [2015-2022] | 2014 [2008-2019] | <0.001 |
| Tumor location |  |  | 0.655 |
| Convexity | 58 (34.5%) | 29 (37.7%) |  |
| Parasagittal | 26 (15.5%) | 9 (11.7%) |  |
| Skull base | 84 (50.0%) | 34 (44.2%) |  |

**Supplementary data 4. Diagnostic ascertainment and validation data.**

|  | **All tumors** | **POM** | **SOM-I** | **SOM-IIA** | **SOM-IIB** |
| --- | --- | --- | --- | --- | --- |
| Total tumors | 168 | 6 | 37 | 57 | 68 |
| Preoperative CT available | 50 | 2 | 11 | 19 | 18 |
| Surgically resected | 89 | 2 | 9 | 28 | 50 |
| Histopathology available | 89 | 2 | 9 | 28 | 50 |
| Bone specimen available | 26 | 2 | 3 | 10 | 11 |
| Bone invasion pathologically confirmed | 26 | 2 | 3 | 10 | 11 |
| Non-assessable bone pathology | 63 | 0 | 6 | 18 | 39 |
| Imaging-only diagnosis | 79 | 4 | 28 | 29 | 18 |

**Supplementary data 5.** Segmentation quality assessment.

To support the robustness of the manual segmentation step, a subset of 45 tumors from the same dataset had previously been independently segmented by a second investigator. Volumetric agreement was excellent (Pearson r = 0.987; 95% CI, 0.977-0.993), with high spatial overlap according to Dice/Jaccard metrics. Because subtype assignment in the present study was deterministic once masks were defined, these findings provide indirect support for the reproducibility of the pipeline. However, formal inter-rater agreement for the final voxel-compartment classification itself was not specifically assessed and remains to be established.

**Supplementary data 6.** Pairwise comparisons of clinical presentation across VOC subgroups.

| **Symptom at diagnosis** | **p-value^$^** | | |  |
| --- | --- | --- | --- | --- |
| **Incidental** |  | | |  |
| POM vs SOM-I | 1.0 | | |  |
| POM vs SOM-IIA | 1.0 | | |  |
| POM vs SOM-IIB | 0.667 | | |  |
| SOM-I vs SOM-IIA | 1.0 | | |  |
| SOM-I vs SOM-IIB | 0.127 | | |  |
| SOM-IIA vs SOM-IIB | 0.126 | | |  |
| **Neurological focal deficit** |  | |  |  |
| POM vs SOM-I | 0.315 | | |  |
| POM vs SOM-IIA | 0.578 |  |  |  |
| POM vs SOM-IIB | 0.583 | | | |
| SOM-I vs SOM-IIA | 0.442 | | | |
| SOM-I vs SOM-IIB | 0.618 | | | |
| SOM-IIA vs SOM-IIB | 0.820 | | | |
| **Cranial nerve deficit** |  | | | |
| POM vs SOM-I | 0.574 | | | |
| POM vs SOM-IIA | 0.581 | | | |
| POM vs SOM-IIB | 1.0 | | | |
| SOM-I vs SOM-IIA | 0.585 | | | |
| SOM-I vs SOM-IIB | **0.024** | | | |
| SOM-IIA vs SOM-IIB | 0.084 | | | |
| **Epileptic seizure** |  | | | |
| POM vs SOM-I | 1.0 | | | |
| POM vs SOM-IIA | 1.0 | | | |
| POM vs SOM-IIB | 0.337 | | | |
| SOM-I vs SOM-IIA | 0.078 | | | |
| SOM-I vs SOM-IIB | **0.001** | | | |
| SOM-IIA vs SOM-IIB | 0.098 | | | |
| **Increased intracranial pressure** |  | | | |
| POM vs SOM-I | 0.263 | | | |
| POM vs SOM-IIA | 0.404 | | | |
| POM vs SOM-IIB | 0.662 | | | |
| SOM-I vs SOM-IIA | 0.645 | | | |
| SOM-I vs SOM-IIB | **<0.001** | | | |
| SOM-IIA vs SOM-IIB | **0.001** | | | |
| **Exophthalmos** |  | | | |
| POM vs SOM-I | 0.155 | | | |
| POM vs SOM-IIA | 0.579 | | | |
| POM vs SOM-IIB | 1.0 | | | |
| SOM-I vs SOM-IIA | 0.058 | | | |
| SOM-I vs SOM-IIB | **<0.001** | | | |
| SOM-IIA vs SOM-IIB | 0.061 | | | |
| **Subcutaneous mass** |  | | | |
| POM vs SOM-I | 0.067 | | | |
| POM vs SOM-IIA | 0.061 | | | |
| POM vs SOM-IIB | **<0.001** | | | |
| SOM-I vs SOM-IIA | 1.0 | | | |
| SOM-I vs SOM-IIB | **0.005** | | | |
| SOM-IIA vs SOM-IIB | **0.001** | | | |
| ^$^Pairwise comparisons of clinical presentation using Fisher’s exact test | | | |  |

**Supplementary data 7.** Sensitivity analysis restricted to one index tumor per patient.

|  | **Index-tumor cohort (n=149)** | **POM (n=5)** | **SOM-I (n=29)** | **SOM-IIA (n=50)** | **SOM-IIB (n=65)** | **p-value** |
| --- | --- | --- | --- | --- | --- | --- |
| **Clinical parameters** |  |  |  |  |  |  |
| Sex |  |  |  |  |  | 0.154 |
| Female | 132 (89) | 4 (80) | 28 (97) | 46 (92) | 54 (83) |  |
| Male | 17 (11) | 1 (20) | 1 (3) | 4 (8) | 11 (17) |  |
| Age (years), mean (SD) | 56.5 (12.6) | 50.8 (13.2) | 54.6 (10.0) | 58.3 (13.9) | 56.4 (12.6) | 0.580 |
| 5mFI ≥2 | 6 (4) | 0 (0) | 0 (0) | 1 (2) | 5 (8) | 0.351 |
| **Symptom at diagnosis** |  |  |  |  |  |  |
| Incidental | 51 (34) | 2 (40) | 12 (41) | 21 (42) | 16 (25) | 0.161 |
| Neurological focal deficit | 27 (18) | 0 (0) | 7 (24) | 8 (16) | 12 (18) | 0.683 |
| Cranial nerve deficit | 17 (11) | 0 (0) | 6 (21) | 7 (14) | 4 (6) | 0.168 |
| Epileptic seizure | 19 (13) | 0 (0) | 0 (0) | 4 (8) | 15 (23) | 0.007 |
| Signs of raised intracranial pressure | 24 (16) | 0 (0) | 1 (3) | 3 (6) | 20 (31) | <0.001 |
| Exophthalmos | 27 (18) | 0 (0) | 12 (41) | 11 (22) | 4 (6) | <0.001 |
| Subcutaneous mass | 15 (10) | 3 (60) | 4 (14) | 8 (16) | 0 (0) | <0.001 |
| **MRI characteristics at diagnosis** |  |  |  |  |  |  |
| Tumor volume (cm³), mean (SD) |  |  |  |  |  |  |
| Intracranial soft tissue component§ | 17.9 (24.9) | 0.0 (0.0) | 2.8 (2.0) | 11.4 (15.1) | 31.0 (30.3) | <0.001 |
| Osseous component | 19.4 (20.7) | 22.1 (15.6) | 24.1 (19.0) | 27.7 (26.4) | 10.6 (11.8) | <0.001 |
| Regional location, n (%) |  |  |  |  |  | 0.070 |
| Skull base* | 71 (48) | 0 (0) | 18 (62) | 24 (48) | 29 (45) |  |
| Convexity/parasagittal | 78 (52) | 5 (100) | 11 (38) | 26 (52) | 36 (55) |  |
| Brain ooedema |  |  |  |  |  | <0.001 |
| No | 86 (58) | 5 (100) | 27 (93) | 37 (74) | 17 (26) |  |
| Yes | 63 (42) | 0 (0) | 2 (7) | 13 (26) | 48 (74) |  |
| Mass effect£ |  |  |  |  |  | <0.001 |
| No | 106 (71) | 5 (100) | 28 (97) | 42 (84) | 31 (48) |  |
| Yes | 43 (29) | 0 (0) | 1 (3) | 8 (16) | 34 (52) |  |
| Except where indicated, data are numbers of tumors with percentages in parentheses.  §Including dural enhancement and intradural expansion.  *Spheno-orbital, clinoid, orbital roof, jugum sphenoidale, tuberculum sellae, cavernous sinus, petrous part of the temporal bone, clivus.  £Defined by compression, displacement, or deformation of the ventricular system. 5mFI: Modified 5-Item Frailty Index. | | | | | | |

**Supplementary data 8.** Sensitivity analysis restricted to histopathologically confirmed tumors (n=89).

|  | **Histopathology confirmed** | **POM (n=2)** | **SOM-I (n=9)** | **SOM-IIA (n=28)** | **SOM-IIB (n=50)** | **p-value** |  |
| --- | --- | --- | --- | --- | --- | --- | --- |
| **Clinical parameters** |  |  |  |  |  |  |  |
| Sex |  |  |  |  |  | 0.628 |  |
| Female | 78 (88) | 2 (100) | 9 (100) | 25 (89) | 42 (84) |  |  |
| Male | 11 (12) | 0 (0) | 0 (0) | 3 (11) | 8 (16) |  |  |
| Age (years), mean (SD) | 55.8 (12.2) | 54.0 (4.2) | 50.8 (9.4) | 58.5 (13.2) | 55.3 (12.2) | 0.478 |  |
| 5mFI ≥2 | 7 (8) | 0 (0) | 0 (0) | 2 (7) | 5 (10) | 0.880 |  |
| **Symptom at diagnosis** |  |  |  |  |  |  |  |
| Incidental | 16 (18) | 1 (50) | 1 (11) | 5 (18) | 9 (18) | 0.641 |  |
| Neurological focal deficit | 21 (24) | 0 (0) | 5 (56) | 7 (25) | 9 (18) | 0.105 |  |
| Cranial nerve deficit | 15 (17) | 0 (0) | 5 (56) | 6 (21) | 4 (8) | 0.009 |  |
| Epileptic seizure | 17 (19) | 0 (0) | 0 (0) | 4 (14) | 13 (26) | 0.234 |  |
| Signs of raised intracranial pressure | 19 (21) | 0 (0) | 0 (0) | 2 (7) | 17 (34) | 0.007 |  |
| Exophthalmos | 16 (18) | 0 (0) | 4 (44) | 7 (25) | 5 (10) | 0.057 |  |
| Subcutaneous mass | 10 (11) | 1 (50) | 1 (11) | 8 (29) | 0 (0) | <0.001 |  |
| **MRI characteristics at diagnosis** |  |  |  |  |  |  |  |
| Tumor volume (cm³), mean (SD) |  |  |  |  |  |  |  |
| Intracranial soft tissue component§ | 25.8 (29.2) | 0.0 (0.0) | 3.2 (1.6) | 16.4 (18.6) | 36.0 (32.4) | <0.001 |  |
| Osseous component | 19.4 (19.9) | 34.0 (14.8) | 26.7 (14.9) | 30.4 (25.2) | 11.3 (12.9) | <0.001 |  |
| Regional location, n (%) |  |  |  |  |  | 0.196 |  |
| Skull base* | 46 (52) | 0 (0) | 7 (78) | 13 (46) | 26 (52) |  |  |
| Convexity/ parasagittal | 43 (48) | 2 (100) | 2 (22) | 15 (54) | 24 (48) |  |  |
| Brain oedema |  |  |  |  |  | <0.001 |  |
| No | 34 (38) | 2 (100) | 7 (78) | 17 (61) | 8 (16) |  |  |
| Yes | 55 (62) | 0 (0) | 2 (22) | 11 (39) | 42 (84) |  |  |
| Mass effect‡ |  |  |  |  |  | <0.001 |  |
| No | 47 (53) | 2 (100) | 8 (89) | 20 (71) | 17 (34) |  |  |
| Yes | 42 (47) | 0 (0) | 1 (11) | 8 (29) | 33 (66) |  |  |
| Except where indicated, data are numbers of tumors with percentages in parentheses.  § Including dural enhancement and intradural expansion.  *Skull base includes spheno-orbital, clinoid, orbital roof, jugum sphenoidale, tuberculum sellae, cavernous sinus, petrous part of the temporal bone, and clivus.  ‡ Defined by compression, displacement, or deformation of the ventricular system.  5mFI: Modified 5-Item Frailty Index. | | | | | | | |

| **Variable** | **1.5T** | **3T** | **P value** |
| --- | --- | --- | --- |
| Age at diagnosis, years, median [IQR] | 53.00 [47.00-66.00] (n=114) | 58.50 [51.25-65.00] (n=46) | 0.255 |
| Year of diagnosis, median [IQR] | 2019 [2015.00-2022.00] (n=114) | 2018 [2015.00-2021.00] (n=43) | 0.526 |
| Female sex | 104/114 (91.2%) | 40/46 (87.0%) | 0.399 |
| VOC class |  |  | 0.539 |
| POM | 5/114 (4.4%) | 1/46 (2.2%) |  |
| SOM-I | 28/114 (24.6%) | 9/46 (19.6%) |  |
| SOM-IIA | 40/114 (35.1%) | 14/46 (30.4%) |  |
| SOM-IIB | 41/114 (36.0%) | 22/46 (47.8%) |  |
| Skull base location | 59/114 (51.8%) | 20/46 (43.5%) | 0.385 |
| Intradural volume, cm³, median [IQR] | 6.78 [1.78-19.80] (n=114) | 5.78 [2.01-19.60] (n=45) | 0.997 |
| Osseous volume, cm³, median [IQR] | 12.19 [6.24-21.40] (n=114) | 11.15 [5.10-21.05] (n=46) | 0.377 |
| Peritumoral oedema on FLAIR |  |  | 0.475 |
| None | 68/114 (59.6%) | 31/46 (67.4%) |  |
| Moderate | 33/114 (28.9%) | 9/46 (19.6%) |  |
| Important | 13/114 (11.4%) | 6/46 (13.0%) |  |
| Midline mass effect | 28/114 (24.6%) | 13/46 (28.3%) | 0.690 |
| Raised ICP / headaches | 14/114 (12.3%) | 9/45 (20.0%) | 0.220 |
| Seizures at diagnosis | 13/114 (11.4%) | 6/45 (13.3%) | 0.788 |
| Exophthalmos | 21/114 (18.4%) | 8/46 (17.4%) | 1.000 |
| Except where indicated, data are numbers of tumors with percentages in parentheses | | | |

**Supplementary data 9.** Sensitivity analysis according to MRI field strength.

**Supplementary data 10.** Unadjusted effect sizes for the main prespecified clinicoradiological associations.

| **Comparison** | **Outcome** | **Exposed group** | **Reference group** | **Crude OR** | **95% CI** | **p-value** |
| --- | --- | --- | --- | --- | --- | --- |
| SOM-IIB vs others | Brain oedema | 49/68 (72.1%) | 16/100 (16.0%) | 13.54 | 6.38-28.74 | <0.001 |
| SOM-IIB vs others | Epileptic seizure | 15/68 (22.1%) | 6/100 (6.0%) | 4.43 | 1.62-12.11 | 0.007 |
| SOM-IIB vs others | Signs of raised intracranial pressure | 21/68 (30.9%) | 6/100 (6.0%) | 7.00 | 2.65-18.51 | <0.001 |
| SOM-I vs others | Exophthalmos | 14/37 (37.8%) | 16/131 (12.2%) | 4.38 | 1.88-10.19 | 0.001 |

**Supplementary data 11.** Sensitivity of voxel-compartment classification to the thickness of the juxta-osseous/dural-proximity layer.

| **Measure** | **5-mm layer, n (%)** | **3-mm layer, n (%)** | **2-mm layer, n (%)** | **Cross-threshold stability / comment** |
| --- | --- | --- | --- | --- |
| **Subtype distribution** |  |  |  |  |
| POM | 6 (3.6) | 6 (3.6) | 6 (3.6) | No reclassification across thresholds |
| SOM-I | 37 (22.0) | 29 (17.3) | 19 (11.3) | Stepwise decrease with thinner layers |
| SOM-IIA | 57 (33.9) | 64 (38.1) | 71 (42.3) | Stepwise increase with thinner layers |
| SOM-IIB | 68 (40.5) | 69 (41.1) | 72 (42.9) | Slight increase with thinner layers; no downward reclassification |
| **Total** | **168 (100)** | **168 (100)** | **168 (100)** |  |
| **Reclassification across thresholds** |  |  |  |  |
| Same subtype: 5 mm vs 3 mm | — | — | — | 159/168 (94.6%) |
| Same subtype: 3 mm vs 2 mm | — | — | — | 155/168 (92.3%) |
| Same subtype: 5 mm vs 2 mm | — | — | — | 146/168 (86.9%) |
| Same subtype across all 3 thresholds | — | — | — | 146/168 (86.9%) |
| Main reclassification pattern | — | — | — | Predominantly SOM-I → SOM-IIA, and less frequently SOM-IIA → SOM-IIB |
| Extreme-class stability | — | — | — | No POM or SOM-IIB tumor changed classification |
| A thinner juxta-osseous/dural-proximity layer led to a progressive reduction in SOM-I cases, with corresponding increases in SOM-IIA and, to a lesser extent, SOM-IIB. Reclassifications were limited to adjacent categories. | | | | |
